# Qatar dental student perceptions of Sirona prep-check software for learning crown preparations

**DOI:** 10.1186/s12909-024-06412-z

**Published:** 2024-12-03

**Authors:** Hanin Daas, María Arregui, Lluís Giner Tarrida, Rebecca Glanville, Kamran Ali

**Affiliations:** 1 College of Dental Medicine, QU Health, Qatar University, Doha, Qatar; 2 Faculty of Dentistry, Universitat Internacional de Catalunya, Barcelona, Spain; 3 Faculty of Health, University of Plymouth, Peninsula Medical School, Faculty of Health, Plymouth, PL4 8AA UK

**Keywords:** Dental education, prepCheck software, Crown preparation, Digital tools, Undergraduate dental students, Human guidance, Pedagogical integration

## Abstract

**Background:**

Contemporary dental education requires swift assimilation of technological advancements to prepare the future generation of dentists. Integrating digital tools, such as prepCheck software in crown preparations offers a promising avenue for enhancing the learning experiences of dental students. This study aimed to evaluate the perceptions and experiences of undergraduate dental students regarding the use of PrepCheck software for learning crown preparations.

**Methods:**

An interventional study design was employed to investigate the perceptions of undergraduate dental students regarding the use of Sirona prepCheck software their learning experiences in the prosthodontics course at the College of Dental Medicine. were recruited using purposive sampling. Participants received training on crown preparations using standard didactic and practical teaching methods. A total of 64 dental students (Mean age 22.4 years) participated in the study. They were randomly assigned to two groups, 32 participants each. The study group utilized prepCheck software and the control group relied solely on supervisor feedback. Both groups completed their crown preparation labs concurrently, ensuring consistency in training and assessment conditions.

**Results:**

Out of a total number of 66 students enrolled on the course, 64 participated in the study giving a response rate of 96.96%. The overall mean score for all items was 1.01 (95%CI 0.77–1.25), indicating positive perceptions of the participants about prepCheck software. Analysis of variance revealed no significant variation by gender. Thematic analysis of open-ended items identified key themes related to the advantages and challenges of using prepCheck. Advantages included immediate self-evaluation, objective analysis, and feedback, while challenges included operational difficulties and cost concerns.

**Conclusion:**

This study provides insights into the effectiveness of prepCheck in dental education, offering perspectives derived from both quantitative and qualitative analyses. A majority of the participants recommended that digital assessment should be integrated with conventional methods, underscoring the importance of human input through supervisor feedback. The study demonstrates the potential of prepCheck software in enhancing participants’ learning experiences in crown preparation assessment. While digital tools offer advantages such as self-evaluation and objective analysis, they need to be supported with input and feedback from the supervisors.

**Supplementary Information:**

The online version contains supplementary material available at 10.1186/s12909-024-06412-z.

## Introduction

Contemporary clinical dental practice requires dental clinicians to assimilate technological advancements to improve standards of care [1]. Digitalization in dentistry, now increasingly supported by Artificial intelligence, is revolutionizing clinical dental practice [2]. Increasingly, digital workflows are being deployed to enhance patient assessment, streamline treatment planning, and design and deliver dental restorations and appliances chairside, eliminating the prolonged waiting times often associated with traditional laboratory workflows [3].

Dentists must possess operative skills and underpinning scientific knowledge to provide predictable, reliable, and effective dental care based on the principles of evidence-based dental practice [4]. Prosthodontics is a core specialty of dentistry and involves fabricating a wide range of fixed and removable prostheses to restore missing tooth structure and aesthetics [5]. Provision of dental crown and bridgework represents a high volume of prosthodontic work and requires high manual dexterity, precision, and hand–eye coordination to restore the dentition following loss of tooth structure or premature loss of teeth. Dentists are expected to use the most conservative options to restore missing tooth structure whilst protecting the dental pulp, maintaining periodontal health, and avoiding damage to hard and soft tissue structures [6].

Crown preparations are often perceived to be one of the most challenging skills in undergraduate dental education and require students to demonstrate a good understanding of the principles of tooth preparation, and manual dexterity [7]. Students often require consolidation of their skills through repeated practice in simulated dental learning environments. Close supervision and objective feedback by faculty are essential in helping students develop the skills required for dental procedures like crown preparation. Faculty employ active coaching and technique demonstrations to ensure students grasp procedures thoroughly [8]. Live and video demonstrations on procedural skills enable students to observe and reinforce techniques [9], while step-by-step instruction with direct observation and immediate feedback provides continuous guidance [10, 11]. Additionally, self-assessment and questioning techniques foster critical thinking and self-reflection, encouraging students to analyze their own performance [12]. Regular structured meetings and motivational commentary also support students’ confidence, progress, and professional development. This comprehensive supervision approach, supported by empirical research, leads to higher achievement of learning outcomes and student satisfaction [13].

Notwithstanding the numerous benefits of faculty supervision to support student learning in simulated dental learning environment, it poses significant resource implications for dental schools to ensure availability of appropriately trained faculty for extended periods in the simulation laboratories. Faculty supervision may be affected by factors such as the halo effect, subjectivity related human judgment errors, and the challenges of supervising multiple students simultaneously [14].

PrepCheck (Dentsply Sirona, Bensheim, Germany) is an objective preparations assessment system designed to document and analyze students’ preparations, offering a supportive tool for enhancing their motor skills crucial in dentistry [15]. Combining Primescan or Omnicam with prepCheck analysis software on the CEREC AC platform allows for comprehensive evaluation and subsequent retrieval of results [16]. Initially introduced at the University of Groningen in the Netherlands, prepCheck employs geometric analysis functions to assess various components of crown preparations including preparation taper, occlusal reduction, axial reduction, preparation type, margin, undercuts, and surface quality [17]. Integration and optimization of learning outcomes using Computer Aided Learning (CAL) alongside the traditional methods of teaching is beneficial [18]. Previous studies on the application of prepCheck crown preparations have highlighted several advantages of this technology and a positive impact on the learning experiences of undergraduate dental students [19].

The conceptual framework for this study draws upon the epistemological tripod of technology education. This perspective examines the application of technology in education through three main lenses: the acquisition of technological scientific knowledge, the development of technical skills, and socio-ethical technical understanding [20]. This heuristic framework serves as a conceptual model, enabling a deeper comprehension of the integration of technology and education. Digital tools in dental education can be tailored to manage the development of technological literacy in dental learning environments and encompass the social interactions therein.

This study aimed to evaluate the perceptions and experiences of undergraduate dental students regarding the use of PrepCheck software for learning crown preparations.

## Materials and Methods

### Ethics approval

The Institutional Review Board of Qatar University approved the study protocol and participant consent forms (Reference number: QU-IRB 1652-EA/22). Before the study, participants were briefed about the purpose and scope of the study. The participation was voluntary, and students were informed that participation in the study would not affect their training or assessment in any form or manner. All participants signed a written consent form.

### Study settings

Qatar University, College of Dental Medicine.

### Study design

An interventional study design was employed to explore the impact of prepCheck, digital assessment tools on student learning, and supervisor teaching practices in dental education.

### Sampling technique and recruitment of participants

Undergraduate dental students enrolled in the fixed prosthodontics course were recruited using purposive sampling. Potential participants received invitations to join the research via email, which included a participant information sheet with a description of the purpose and scope of the study.

### Sample size calculation

The sample size for this study was determined using a power analysis with G*Power software (version 3.1) [21]. The estimated sample size was based on using an independent group’s t-test to compare the mean performance of two groups (control group, CG and study group SG), with a two-tailed hypothesis anticipating a medium effect size (*d* = 0.5, based on existing literature), and conducting significance testing at *α* = 0.5, whilst maintaining a power level of 1-*β* = 0.8. This study aimed to explore the perceptions and experiences of undergraduate dental students when their crown preparations are assessed digitally compared to conventional feedback and assessment by their supervisors. The number of completed crown preparations served as a sample size determinant to ensure that dental students in both the study and control groups had comparable levels of hands-on experience. This approach was designed to provide an equivalent experiential base, enabling students to accurately communicate their perceptions of the learning methods. Accordingly, the minimum sample required was 128 crown preparations divided equally between the two groups. These parameters balance recruitment demands and the risk of Type-I and Type-II errors.

### Data collection instrument

A questionnaire consisting of eleven closed and three open-ended items was drafted by a team of five experienced clinical dental academics (supplementary material 1). The face and content validity of the items were determined by mapping the questionnaire items to the learning outcomes of the prosthodontic curriculum related to crown preparation.

The closed-ended questions were scored on a Likert scale consisting of five categories: Strongly disagree (-2); Disagree (-1); Unsure (0), Agree (1); Strongly Agree (2). Three open-ended questions were incorporated, enabling participants to express their opinions regarding the strengths and weaknesses of prepCheck and offer suggestions for enhancing student learning experiences in crown preparation assessment.

The pretesting of the questionnaire was carried out in line with established practices. Undergraduate dental students (*N* = 6), and dental academics (*N* = 3) participated in pretesting of the questionnaire electronically. The purposes of pretesting of scale items were as follows:

1. Determine the content and face validity of scale items.
2. Determine the clarity of the scoring categories.
3. Determine the participants’ clarity and consistent interpretation of the questionnaire.
4. Determine the correlations between ordinal variables

Pre-testing the questionnaire showed that the scale effectively captured the intended measurements of the study. Feedback indicated the need for minor language adjustments for two items before finalizing the questionnaire for administration. Kendall’s Tau showed satisfactory correlations between ordinal variables in closed ended items (*τ* = 0.76).

### Data collection

**Fig. 1:**
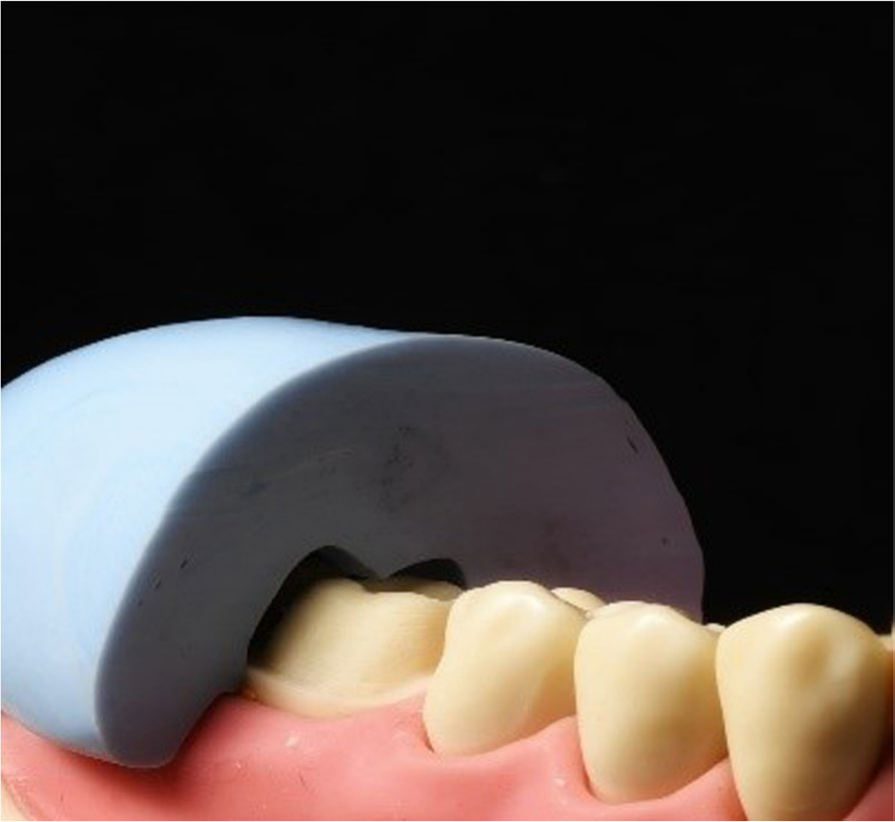
Use of putty index to assess the depth of the crown preparation

All participants received core training on crown preparations for full ceramic crowns on a maxillary central incisor and a mandibular first molar using standard didactic and practical teaching. Artificial teeth by Frasaco (Frasaco, Tettnang, Germany) mounted on a Frasaco model in a mannequin on a dental simulation unit (Sirona Dental GmbH), were used throughout. Before starting preparation, each participant prepared a silicone putty index (3 M Inc., USA) of the teeth to assess the depth of the tooth reduction as depicted in Fig. 1.

**Fig. 2:**
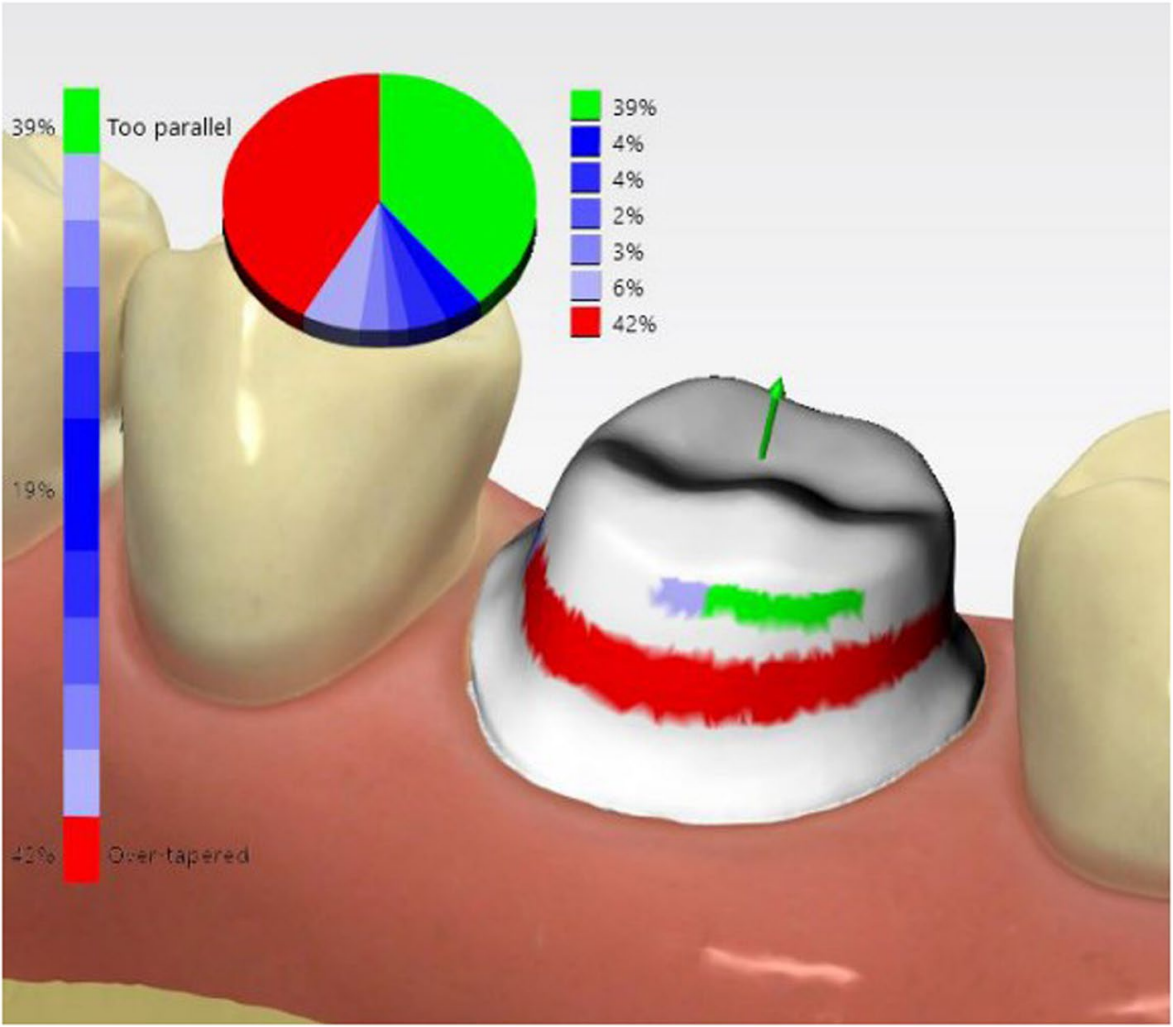
PrepCheck digital assessment tool for crown preparation

All participants also attended a demonstration on digital assessment for crown preparation using prepCheck software (prepCheck 5.0.x PRO, Dentsply Sirona, Bensheim, Germany) as depicted in Fig. 2.

All participants received core training on crown preparations for full ceramic crowns on a maxillary central incisor and a mandibular first molar using standard didactic and practical teaching. Artificial teeth by Frasaco (Frasaco, Tettnang, Germany), mounted on a Frasaco model in a dental mannequin (Sirona Dental GmbH), were used throughout. Before starting the preparation, each participant created a silicone putty index (3 M Inc., USA) of the teeth to assess the depth of tooth reduction, as shown in Fig. 1. Additionally, all participants attended a demonstration on digital assessment for crown preparation using PrepCheck software (prepCheck 5.0.x PRO, Dentsply Sirona, Bensheim, Germany), as illustrated in Fig. 2.

Following standardized training in crown preparation techniques, participants were randomly assigned to two groups: the study group, which used PrepCheck-enhanced training, and the control group, which used usual training with supervisor feedback. Each group attended four laboratory sessions (three hours each), covering crown preparations for a maxillary central incisor and a mandibular first molar. Both groups trained in parallel: the PrepCheck group conducted self-assessments using the software, while the control group received only supervisor feedback. This concurrent training schedule ensured a fair comparison between digital and conventional feedback approaches.

Following standardized training in crown preparation techniques, participants were randomly assigned to two groups to evaluate the instructional efficacy of digital assessment tools.

#### Group 1 (Study Group)

**Fig. 3:**
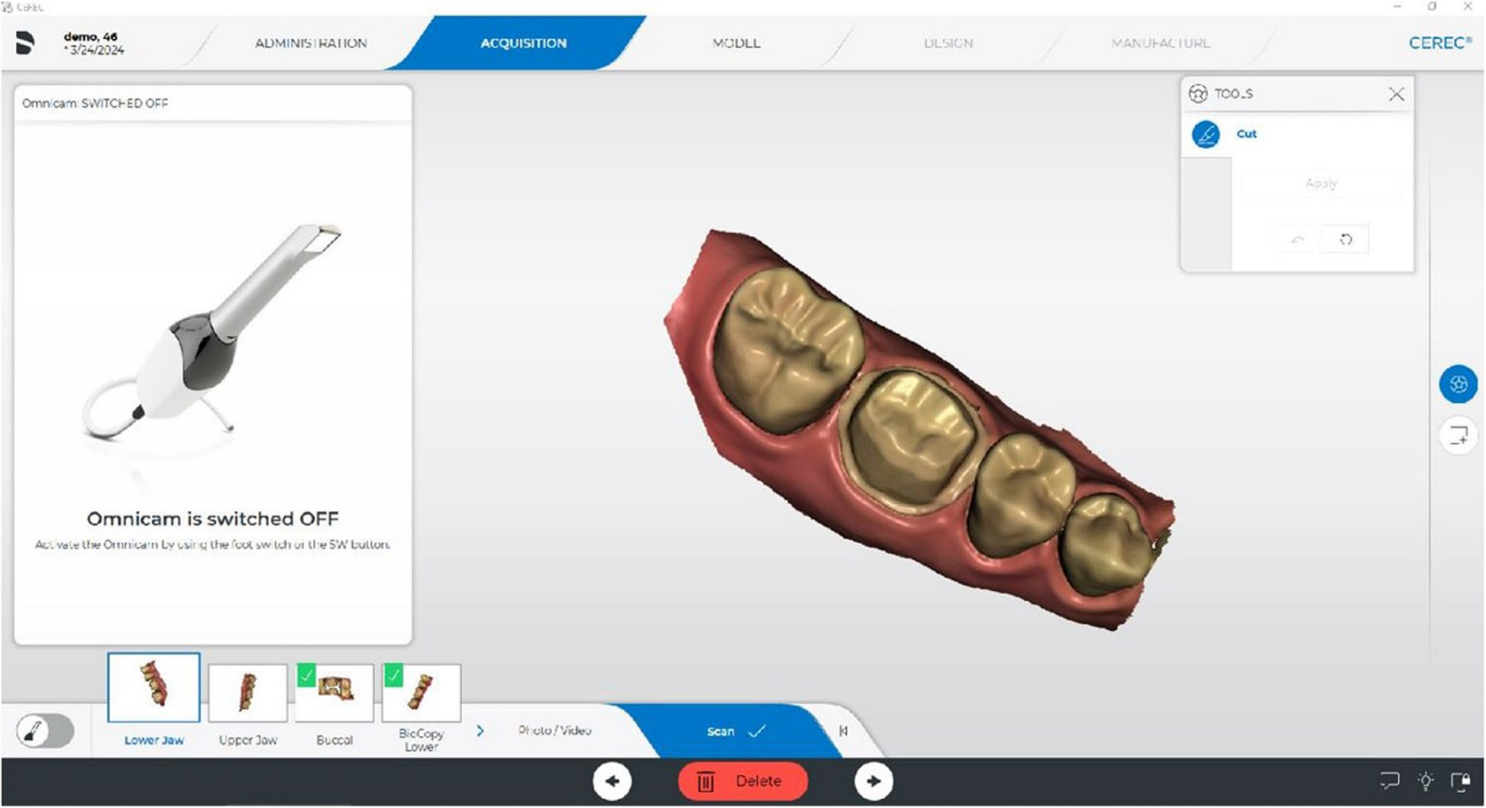
Preparation scan along with the adjacent teeth, antagonists, and bite

The participants in the study group carried out 64 crown preparations and they assessed their preparation using prepCheck software. The tasks included one crown preparation on the maxillary incisor and one molar for each participant. They consolidated their crown preparation skills over four sessions (3 h each); two sessions for full ceramic crown preparation on a maxillary central incisor and two sessions for a mandibular first molar. The CEREC Omnicam intra-oral scanner (Dentsply Sirona, Bensheim, Germany) was utilized for the scanning process, and digital scans were performed using CEREC software SW 5.1 (Dentsply Sirona, Bensheim, Germany). To perform the digital preparation analysis with prepCheck, participants in the study group selected the tooth and the type of restoration (full crown). They scanned the preparation along with the adjacent teeth, antagonists, and bite using the Omnicam intra-oral scanner (Fig. 3). Subsequently, they defined the preparation margin and the insertion axis.

The scanned data were imported into prepCheck software version 5.0.x PRO. The participants self-assessed their crown preparation using prepCheck, comparing their preparation to pre-set parameters defined by faculty supervisors, and uploaded it on the prepCheck wizard. The differences were displayed visually using a color scale and shown by measured angles and metric values. Participants could view the models from all directions and use the zoom function for a more detailed view. The automatic analysis functions included undercut, taper, reduction occlusal, reduction axial preparation type, margin, surface quality, and preservation of adjacent teeth.

#### Group 2 (Control Group)

The participants in the control group carried out 64 crown preparations following a parallel training regimen but only relied on supervisor feedback, without access to prepCheck software. Participants consolidated their crown preparation skills over four sessions (3 h each); two sessions for full ceramic crown preparation on the maxillary central incisor and two sessions for the mandibular first molar.

### Formative assessment

Participants from both groups carried out full ceramic crown preparations on a maxillary central incisor, and a mandibular first molar tooth in two separate sessions (one session for the upper central incisor and the other one for lower first molar crown preparation). The supervisors utilized the standard assessment rubric used by the institution for ceramic crown preparations. The assessors who evaluated the crown preparations were distinct from the supervisors who were present with the students during practice sessions. This separation ensured that the assessors remained blinded throughout the evaluation process, allowing them to focus solely on assessing the crown preparations without any knowledge of the participants’ group affiliations.

Finally, after the assessment, the participants in the control group were allowed to experience their crown preparation digital assessment through prepCheck software similar to their study group counterparts. Similarly, the study group also received additional sessions with supervisors to benefit from their feedback.

### Data collection

Once all participants had experienced using both digital assessments with PrepCheck and conventional visual assessments by the supervisors, they were invited to participate in an online questionnaire via Google Forms to record their experiences and perceptions. Before accessing the questionnaire, participants were required to sign an electronic consent form, confirming their understanding of the study’s purpose, the voluntary nature of their participation, and the anonymous handling of their data.

### Data analyses

All data were analyzed and visualized using RStudio (version 2023.06.2) incorporating R version 4.0.5. The normality of the data was confirmed with the Shapiro–Wilk Test. The quantitative questionnaire comprised eleven items, which were positively phrased and so positively scored. Descriptive statistics including confidence intervals were calculated for each item and the combined dataset. Analysis of Variance was used to determine any significant variation between the results by gender. Estimated marginal means were calculated from the ANOVA outcomes.

## Results

Out of a total number of 66 students enrolled on the course, 64 participated in the study giving a response rate of 96.96%. The participants included 47 females (73.43%) and 17 males (26.56%). Although the students were divided into two groups, all students experienced the use of PrepCheck software and supervisor feedback, albeit at different times. Therefore, the mean score for all eleven closed-ended items was calculated for all participant. Descriptive values for each item are summarized in Table. The mean score for all items combined was 1.01, with a 95% confidence interval ranging from 0.77 to 1.25. This indicates a generally positive perception and perceived effectiveness of the digital assessment tools used in the study (Table 1).

**Table 1:** Descriptive values (all respondents)

Item description	Mean	St. Dev	95% CI (lower)	95% CI (upper)
1.The instructions for PrepCheck were clear	0.92	0.98	0.68	1.17
2. Self-evaluation during crown preparation is helpful	1.15	0.95	0.92	1.38
3. Self-evaluation of my crown preparation on the monitor using an objective analysis with prepCheck improves my understanding	0.98	1.05	0.73	1.24
4. PrepCheck helps improve the quality of my crown preparations	1.03	0.96	0.79	1.27
5. Using prepCheck, evaluation of the finished preparation is easy	0.95	1.03	0.7	1.21
6. PrepCheck provides an objective assessment of crown preparations	1.08	0.83	0.87	1.28
7. The feedback from faculty members on the crown preparations is consistent	0.64	1.08	0.37	0.9
8. The assessment by faculty members is fair	0.92	0.85	0.72	1.13
9. Conventional assessment by faculty members helps identify my learning needs	1.26	0.81	1.06	1.46
10. Faculty members should routinely use PrepCheck to assess the crown preparations on the prosthodontic course	0.85	1.07	0.59	1.11
11. PrepCheck is more appropriate as a supplementary tool rather than a stand-alone method for the evaluation of crown preparations	1.35	0.92	1.12	1.57
Overall	1.01	0.97	0.77	1.25

Descriptive values for each item, by gender, are shown in Table 2.

**Table 2:** Descriptive values by gender

	**Mean**		**Standard Dev**		**95% CI (lower)**		**95% CI (upper)**	
**Question Id**	**Female**	**Male**	**Female**	**Male**	**Female**	**Male**	**Female**	**Male**
Question1	0.92	0.94	1.02	0.9	0.63	0.51	1.21	1.38
Question2	1.20	1.00	0.93	1.00	0.94	0.52	1.47	1.48
Question3	1.00	0.94	1.02	1.14	0.71	0.39	1.29	1.50
Question4	1.04	1.00	0.93	1.06	0.77	0.49	1.31	1.51
Question5	1.00	0.82	1.02	1.07	0.71	0.30	1.29	1.34
Question6	1.04	1.18	0.84	0.81	0.80	0.78	1.28	1.57
Question7	0.55	0.88	1.14	0.86	0.23	0.47	0.88	1.30
Question8	0.90	1.00	0.82	0.94	0.66	0.55	1.13	1.45
Question9	1.29	1.18	0.68	1.13	1.09	0.63	1.48	1.72
Question10	0.82	0.94	1.11	0.97	0.50	0.47	1.13	1.41
Question11	1.41	1.18	0.81	1.19	1.18	0.60	1.64	1.75
*Overall*	1.01	1.01	0.97	0.99	0.78	0.76	1.25	1.25

The ANOVA did not show any statistically significant differences in the mean scores based on gender (*p* = 0.908), and both groups had an adjusted mean of 1.01 for all items.

### Responses to open-ended questions

A thematic analysis was carried out to analyze the responses to open-ended questions. The key themes related to the advantages and challenges of using prepCheck are summarized in Table 3. The results showed the main benefits were the opportunities for self-evaluation and timesaving. However, participants also highlighted the importance of supervisor, feedback to enhance their learning and improve translation of pre-clinical skills into clinical settings.

**Table 3:** Advantages and challenges of using PrepCheck for crown preparation assessment

Theme	Subtheme	Responses	Score^a^
1-Advantages of Using Software for Dental Crown Preparations	Self-evaluation	The software allows immediate self-evaluation Errors/deficiencies in tooth preparation were identified immediately	+ + +
	Timesaving	Feedback when required without the need to wait for the supervisors	+ + +
	Objective analysis and feedback	Consistent analysis and feedback Less chance of subjectivity Minimal risk of a halo effect	+ +
2- Limitations of Using prepCheck for evaluation of crown preparations	Operational difficulties	The initial learning curve to operate the software effectively Software may be difficult to operate and pose technical issues such as lags and crashes Delays due to technical issues	+ +
	Cost concerns	Concerns were raised regarding the cost of implementing and maintaining the software across multiple stations in the laboratory	+ +
3-Adequacy of using prepCheck software for assessment of crown preparation	Preference for additional feedback from supervisors	Additional feedback from supervisors alongside prepCheck analysis may provide a more comprehensive evaluation Participants highlighted the importance of human input for insights and comprehensive assessment, underscoring the value of supervisor feedback	+ + +

^a^Each + represents responses from 20% participants

While PrepCheck software provided precise and objective assessments of crown preparations, participants valued the nuanced feedback and expertise that supervisors offered. This combination was seen as crucial for a comprehensive learning experience, ensuring that participants not only understood the technical aspects but also gained insights into clinical judgment and decision-making. Initial training sessions on the PrepCheck software were pivotal in enhancing participants’ confidence and competence. The majority expressed that using the PrepCheck software in conjunction with teacher guidance, rather than as a standalone tool, was more beneficial. This approach allowed participants to immediately apply digital feedback within the context of real-time supervision, facilitating a deeper understanding and immediate rectification of errors. Regarding the cost of implementing PrepCheck software, participants expressed a range of opinions. While some acknowledged the financial investment required, many felt that the benefits in terms of enhanced feedback, precision, and the potential for improved clinical skills justify it.

## Discussion

Clinical dental practice continues to be influenced by technological advancements, with a noticeable rise in the application of digital technology to enhance treatment outcomes and patient experiences. These developments reflect a growing trend towards the digitalization of dentistry, which needs to be mirrored in dental education to enhance student-learning experiences and better prepare them for contemporary dental practice [14]. Digital technology and virtual reality dental simulation with haptic feedback are being increasingly employed in undergraduate dental education to enhance the learning experiences of dental students [22–24]. To the best of our knowledge, this is the first study on the use of prepCheck software by undergraduate dental students in the Middle East.

Training in tooth preparation is crucial to enhance their skills and minimize errors in clinical settings and digital dentistry can serve as a valuable tool to enhance the psychomotor skills of dental students [25]. Digital PrepCheck software has been used for training dental students on crown preparations, and cavity design. Wolgin et al*.,* (2018) reported its use in designing macro-retentive Class II cavity preparations in a study involving 54 students, and the software allowed precise evaluations of students’ practical skills and will aid for accurate assessment of macro-retentive preparations [26].

Introduction of digital technology into pre-clinical training can potentially boost dental students; engagement and improve the quality of student assessments with a more objective assessment of tasks performed by the student in dental simulation laboratory. For this, feedback by supervisors using conventional visual assessments is increasingly being augmented or even substituted by digital tools [1]. However, it is essential to critically evaluate the benefits and limitations of digital tools to provide the most appropriate learning experiences to the students. The results of this study corroborate with previous research studies and underscore the importance of using both conventional and digital methods together; yielding successful outcomes for students [15, 17, 27–30].

Self-assessment is a recognized tool to enhance critical evaluation and reflective practice amongst students. With appropriate training, digital prepCheck software can be utilized effectively by the students to self-assess their psychomotor skills in simulated dental environments and improve the quality of their work. Despite the initial investment costs in digital technology, it can minimize the reliance on direct supervision by the dental faculty and potentially contribute to cost savings in the long term [31]. This study did not undertake a cost–benefit analysis of digital technology but could be examined in future research.

Mixed perceptions of students regarding the use of digital tools in dental education are reported in the literature. Kwon et al. (2015) evaluated the impact of computer-assisted learning on the waxing abilities of dental students in the U.S, comparing software grading with faculty evaluations. Their findings indicated that students viewed digital technology less favorably [32]. Another study reported differences in the grades given by supervisors compared to those from PrepCheck technology, concluding that supervisor grades were inflated, which may confuse the students [33]. Direct communication and interaction between supervisors and students are crucial for assessing dental crown preparations. Such interactions provide immediate feedback, address doubts, and offer personalized guidance, creating an environment conducive to learning and active student participation [17]. However, conventional methods rely heavily on supervisors, with potential influences from factors such as the halo effect, available time, and human judgment errors [14]. The findings of the current study offer a more balanced perspective and whilst the participants shared positive experiences with prepCheck software, they also recognized the importance of supervisor input and feedback to support their learning in crown preparations. Notwithstanding the benefits of supervisor feedback, there was a consensus on the benefits of PrepCheck software, which provided immediate, focused, and objective feedback. These findings support the use of digital technology in education to enhance student learning capabilities [18]. Challenges related to learning the software and technical difficulties can be addressed through additional training and experience.

Whilst digital technology is becoming increasingly established in dental education, its value in summative assessment of students remains to be determined. Previous studies comparing the outcomes of digital assessments with conventional methods show mixed results.

This study provides valuable insights that can significantly advance educational practices in dentistry by incorporating innovative digital tools. However, it is important to acknowledge some limitations. The study sample was drawn from a single institution, which may limit the generalizability of the findings. Additionally, the relatively small sample size might not fully capture the diverse experiences and perceptions of the broader student population. Further multi-center studies based on a larger sample size are recommended to determine the value of digital assessment of crown preparations in undergraduate dental curricula. Despite these limitations, the integration of digital tools like PrepCheck in dental education has shown to be beneficial. Digital assessments enhance student learning by providing precise, objective feedback that helps identify areas for improvement. However, a combination of digital and conventional methods appears to offer the most comprehensive educational outcomes.

## Conclusion

This study provides insights into the perceptions and experiences of undergraduate dental students regarding the use of PrepCheck software. Although the participants valued the PrepCheck software for its objectivity and consistency, the majority of the participants expressed the desire for additional feedback from the supervisors to enhance their learning. As with many other skills, both the conventional and digital tools have their pros and cons in dental education and both methods should be used to maximize the learning opportunities for dental students and enhance their psychomotor skills. Further multi-center studies using a larger sample size are recommended to enhance the generalizability of the findings.

## Supplementary Information

Supplementary Material 1.

## Data Availability

Underlying data available from the corresponding author on a reasonable request.
