# Supplementary material for: Qatar dental student perceptions of Sirona prep-check software for learning crown preparations
Source: BMC Med Educ. 2024 Dec 3;24:1409. doi: 10.1186/s12909-024-06412-z (PMC11616169; doi:10.1186/s12909-024-06412-z)
Supplement: Supplementary file 1 — Supplementary Material 1. [file 12909_2024_6412_MOESM1_ESM.docx]

**Supplementary Material 1: Study Questionnaire**

| **Closed-ended Questions** | **Strongly disagree (-2)** | **Disagree (-1)** | **Unsure (0)** | **Agree (1)** | **Strongly Agree (2)** |
| --- | --- | --- | --- | --- | --- |
| 1- The instructions for PrepCheck were clear |  |  |  |  |  |
| 2- Self-evaluation during crown preparation is helpful |  |  |  |  |  |
| 3- Self-evaluation of my crown preparation on the monitor using an objective analysis with prepCheck improves my understanding |  |  |  |  |  |
| 4- PrepCheck is helpful to improve the quality of my crown preparations |  |  |  |  |  |
| 5- Using PrepCheck, evaluation of the finished preparation is easy |  |  |  |  |  |
| 6- PrepCheck provides an objective assessment of crown preparations |  |  |  |  |  |
| 7- The feedback by faculty members on crown preparations is consistent |  |  |  |  |  |
| 8- The assessment by faculty members is fair |  |  |  |  |  |
| 9- Conventional assessment by faculty members is helpful to identify my learning needs |  |  |  |  |  |
| 10- Faculty members should routinely use PrepCheck to assess the crown preparations on the prosthodontic course |  |  |  |  |  |
| 11- PrepCheck is more appropriate as a supplementary tool rather than a stand-alone method for evaluation of crown preparations |  |  |  |  |  |

***Open ended Questions***

1. In your opinion what are the main advantages of using prepCheck software for evaluation of crown preparations?
2. What are the main limitations of using prepCheck for evaluation of crown preparations?
3. Is crown preparation assessment using the analysis tools provided by prepCheck adequate or would additional feedback by the supervisor be required? Please explain
